# Der Sterbeort älterer Menschen mit einer Demenz: Epidemiologische Daten einer Beobachtungsstudie zu Sterbeorten in Deutschland (2001, 2011, 2017)

**DOI:** 10.1007/s00391-021-01976-7

**Published:** 2021-09-30

**Authors:** Burkhard Dasch, Philipp Lenz

**Affiliations:** Zentrale Einrichtung Palliativmedizin, Universitätsklinikum Münster, Albert-Schweitzer-Campus 1, Gebäude W30, 48149 Münster, Deutschland

**Keywords:** Demenz, Ältere Patienten, Sterbeort, Todesbescheinigung, Beobachtungsstudie, Dementia, Older patients, Place of death, Death certificate, Observational study

## Abstract

**Hintergrund:**

Die Demenz wird aufgrund ihres progredienten Krankheitsverlaufes mit verkürzter Lebenserwartung und oft fehlender therapeutischer Möglichkeit einer Kuration zunehmend als terminale Erkrankung wahrgenommen. Im palliativmedizinischen Kontext gilt der präferierte Sterbeort als Qualitätsindikator für eine bedarfsgerechte Patientenversorgung. Ziel der vorliegenden Studie war es, die Verteilung des Sterbeortes für ältere Menschen mit einer demenziellen Erkrankung zu beschreiben.

**Material und Methode:**

Datengrundlage bildeten ausgewertete Todesbescheinigungen der Jahre 2001, 2011, 2017 der bis dato umfangreichsten Sterbeortstudie in Deutschland, die in ausgewählten Regionen Westfalens durchgeführt wurde. Ärztliche Angaben zur Todesursache wurden miterfasst. So konnten Verstorbene ≥ 65 Jahre mit einer Demenzerkrankung (ICD-10: F01, F02, F03, G30) identifiziert und deren Sterbeort deskriptiv dargestellt werden.

**Ergebnisse:**

Eine Demenzerkrankung lag bei Verstorbenen ≥ 65 Jahre (*n* = 31.631) in 14,9 % [95 %-KI: 14,5–15,3 %] der Fälle vor. Es ergab sich folgende Sterbeortverteilung (%, altersstandardisiert; 2001/2011/2017): häusliches Umfeld 24,0/19,7/15,8; Krankenhaus 40,4/29,0/24,3; Palliativstation 0,0/0,3/1,8; Hospiz 0,4/0,9/0,9; Pflegeheim 35,2/49,5/57,1; sonstiger Ort 0,0/0,0/0,0.

**Schlussfolgerung:**

Ältere Menschen (≥ 65 Jahre) mit Demenz versterben mehrheitlich im Pflegeheim, gefolgt vom Krankenhaus und vom häuslichen Umfeld. Palliativstationen und Hospize spielen als Sterbeorte bei diesen Personen eine untergeordnete Rolle.

**Zusatzmaterial online:**

Zusätzliche Tabellen sind in der Online-Version dieses Artikels (10.1007/s00391-021-01976-7) enthalten.

## Abstract (en)

**Background:**

Dementia is increasingly perceived as a terminal illness due to disease progression with a shortened life expectancy and often a lack of therapeutic options. In the context of palliative care, the preferred place of death is considered a quality indicator for needs-based patient care. The aim of this study was to describe the distribution of places of death of older patients with dementia.

**Material and methods:**

Death certificates from the years 2001, 2011 and 2017 were evaluated from the most comprehensive study on places of death in Germany to date, conducted in selected regions of Westphalia. Medical information on the cause of death was also analyzed. In this way, deceased patients with dementia ≥ 65 years (ICD-10: F01, F02, F03, G30) were identified and the distribution of their places of death statistically determined.

**Results:**

Dementia was present in 4720 out of 31,631 (14.9%; 95% CI: 14.5–15.3%) deceased patients ≥ 65 years. The distribution of places of death was as follows (%; age-standardized; 2001/2011/2017): home environment 24.0/19.7/15.8, hospital 40.4/29.0/24.3, palliative care unit 0.0/0.3/1.8, hospice 0.4/0.9/0.9, nursing home 35.2/49.5/57.1 and other places 0.0/0.0/0.0.

**Conclusion:**

The majority of older patients (≥ 65 years) with dementia die in nursing homes, followed by hospitals and the home environment. Palliative care units and hospices play a subordinate role as places of death for patients with dementia.

In Deutschland leben rund 1,6 Mio. Menschen mit einer Demenz [7]. Die Wahrscheinlichkeit zu erkranken ist mit dem Alter assoziiert und steigt nach dem 65. Lebensjahr steil an. Frauen sind insbesondere aufgrund ihrer höheren Lebenserwartung häufiger betroffen als Männer. Daneben werden aber auch genetische, hormonelle und Lebensstilfaktoren für den Geschlechtsunterschied angeführt [20]. In den meisten Fällen liegen neurodegenerative Erkrankungen und/oder Durchblutungsstörungen des Gehirns zugrunde, wobei die Alzheimer-Krankheit die häufigste Demenzform darstellt [1, 15]. Aktuelle Prognosen erwarten für das Jahr 2050 einen Anstieg demenzieller Erkrankungsfälle in Deutschland auf 2,4–2,8 Mio. [7].

Zumeist schreitet die Erkrankung bis zum Tode unweigerlich fort, wobei Alter, Symptomschwere und Komorbiditäten die Krankheitsdauer mitbestimmen [17]. Im Vergleich zu nichtdementen Personen besitzen Demenzerkrankte ein 2‑ bis 5‑fach höheres Sterblichkeitsrisiko [2]. Im Einzelfall lässt sich jedoch die Krankheitsdauer schwer vorhersagen. Angesichts der limitierten Therapiemöglichkeit, des progredienten Krankheitsverlaufs und der reduzierten Lebenserwartung werden palliativmedizinische Versorgungsaspekte bei Demenzerkrankten in zunehmendem Maße wahrgenommen [9, 19, 28].

In der modernen Hospiz- und Palliativbewegung gilt der präferierte Sterbeort als Qualitätsindikator für eine bedarfsgerechte Versorgung Sterbender. Internationale Sterbeortstudien zur Demenz ergeben ein heterogenes Bild [6, 10, 12, 14, 16, 18, 24–26, 29, 30]. Überwiegend versterben in westlichen Industrienationen demente Personen im Pflegeheim, gefolgt vom Krankenhaus und eher seltener im häuslichen Umfeld.

In Deutschland existieren bislang nur wenige Untersuchungen zum Sterbeort von Menschen mit Demenz. Escobar Pinzon et al. generierten eine Zufallsstichprobe Verstorbener des Jahres 2008 des Bundeslands Rheinland-Pfalz und führten eine Befragung Hinterbliebener durch [10]. Hierbei ergab sich folgende Sterbeortverteilung: häusliches Umfeld 42,2 %, Pflegeheim 26,9 %, Krankenhaus 26,2 %. Der außergewöhnlich hohe Anteil häuslicher Sterbefälle dürfte wahrscheinlich auf einen Selektionsbias zurückzuführen sein. Es ist anzunehmen, dass Hinterbliebene, die eine demenzerkrankte Person bis zu ihrem Tod zu Hause versorgt hatten, häufiger eine Studienrückantwort gaben, vergleichend zu Angehörigen, wo sich der Tod der/des Verstorbenen in einer Institution ereignet hatte.

Die bis dato umfangreichste Sterbeortstudie in Deutschland wurde von Dasch et al. in Westfalen durchgeführt [6]. Fast 40.000 Totenscheine ausgewählter westfälischer Regionen der Jahre 2001, 2011, 2017 wurden ausgewertet. In diesem Kontext wurden auch ärztliche Angaben zur Todesursache erfasst, u. a. die Dokumentation einer Demenz. Nachfolgend werden nun die Studienresultate (2001–2017) der Sterbeortverteilung verstorbener Menschen ≥ 65 Jahre mit Demenz dargestellt.

## Methode

### Design

Das Design der Studie basierte auf 3 Querschnittserhebungen sämtlicher Todesbescheinigungen der Studienregion (2001, 2011, 2017).

### Studienregion

Die Studienregion umfasst die Städte Bochum und Münster sowie die Landkreise Borken und Coesfeld (Westfalen, NRW; Zusatzmaterial online: e‑Tab. 1). Aus Datenschutzgründen musste die Datenerhebung in den jeweiligen Gesundheitsämtern erfolgen.

### Datenanalyse

Für die Analyse wurde der komplette Datensatz aller Todesbescheinigungen der Jahrgänge 2001, 2011, 2017 herangezogen. Folgende Informationen wurden erhoben: Sterbeort, Sterbedatum, Sterbezeit, Wohnort, Alter, Geschlecht, Todesart, ausgewählte Informationen zur Todesursache (u. a. Tumor- und Demenzerkrankung). Die Angaben zum Sterbeort wurden auf Plausibilität überprüft, indem nachverfolgt wurde, ob die Adresse glaubhaft einer Wohnanschrift, einer Institution oder einem öffentlichen Ort zugewiesen werden konnte.

### Verstorbene mit Demenz

Verstorbene Personen mit einem Sterbealter ≥ 65 Jahre, bei denen in der Todesursache gemäß ICD-10 eine Alzheimer-Erkrankung (G30), eine vaskuläre Demenz (F01), eine Demenz bei anderorts klassifizierten Krankheiten (F02) oder eine nicht näher bezeichnete Demenz (F03) beschrieben war, wurden zur Gruppe Demenzerkrankter zusammengefasst. Hierbei wurden sämtliche ärztliche Angaben zur Todesursache ausgewertet (I: Grundleiden, Folge von, unmittelbare Todesursache, II: mit zum Tode führende Krankheiten ohne Zusammenhang mit dem Grundleiden, III: Epikrise).

### Definition des Sterbeortes

Der Sterbeort wurde in die Kategorien häusliches Umfeld, Krankenhaus, Palliativstation, Pflegeheim, Hospiz und sonstiger Ort eingeteilt. Eine Palliativstation zählte als eigenständiger Sterbeort. Zur Kategorie Pflegeheim wurden das Altenheim, das Seniorenheim, Wohnformen des betreuten Wohnens sowie die Kurzzeitpflege gezählt. Sonstige Orte betrafen u. a. öffentliche Plätze, Hausarztpraxen, Freizeiteinrichtungen.

### Statistische Auswertungen

Die Häufigkeit des Sterbeortes sowie Geschlechts- und Altersverteilung Verstorbener wurden absolut und prozentual dargestellt. Ein Trendtest über die Jahrgänge 2001, 2011, 2017 wurde durchgeführt (kategorielle Daten > Chi-Quadrat-Test, stetige Daten > Kruskal-Wallis-Test), und bei signifikantem Ergebnis wurden nachfolgende Tests (2001 vs. 2011, 2001 vs. 2017, 2011 vs. 2017) angeschlossen (kategoriellen Daten > Chi-Quadrat-Test, parametrische Daten > unverbundener *t*-Test, nichtparametrische Daten > Mann-Whitney-U-Test). Um die Erhöhung der α‑Fehler-Wahrscheinlichkeit durch multiples Testen zu minimieren, wurde das Signifikanzniveau *p* < 0,05 einer Bonferroni-Korrektur unterzogen. Um Verzerrungen des Sterbeortes durch Alterseffekte auszugleichen, wurde eine direkte Altersstandardisierung durchgeführt. Als Standardpopulation definierten wir die Studienbevölkerung aller 3 Jahrgänge mittleren Alters. Die Auswertungen erfolgten mit der SPSS-Version 26.

### Ethikvotum und Datenschutz

Unter Wahrung gesetzlich vorgeschriebener Datenschutzbedingungen wurde die Einsicht in archivierte Todesbescheinigungen vonseiten der Gesundheitsämter erlaubt. Die Studie wurde mit Zustimmung der Ethik-Kommission der Ruhr-Universität Bochum (Votum-Nr.: 17-6330) gemäß der Deklaration von Helsinki von 1975 (in der aktuellen überarbeiteten Fassung) sowie im Einklang mit nationalem Recht durchgeführt.

## Ergebnisse

**Figure 1:**
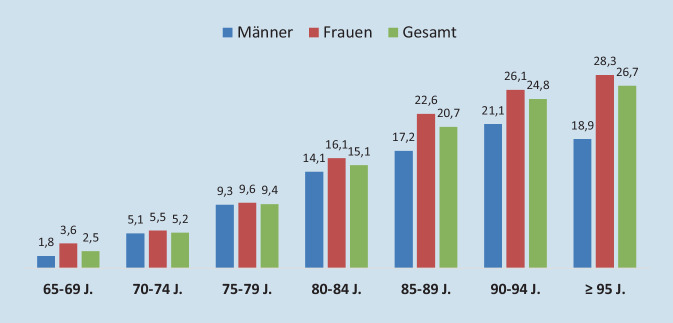

Insgesamt wurden 38.954 Todesbescheinigungen ausgewertet, wobei bei 81,2 % (31.631) das Sterbealter ≥ 65 Jahre lag. In 14,9 % der Fälle [95 %-KI: 14,5–15,3 %] (4720) war eine Demenz dokumentiert worden. Die absolute Zahl von Sterbefällen mit Demenz war von 858 (2001) auf 2111 (2017) angestiegen, entsprechend einer rohen Mortalitätsrate von 350 (2001) bzw. 656 (2017) Fällen pro 100.000. Zwei Drittel der verstorbenen Demenzerkrankten waren Frauen und ein Drittel Männer. Das mittlere Sterbealter lag bei 87,4 (Frauen) bzw. 83,4 (Männer) Jahren (Tab. 1). Die Demenzprävalenz stiegt mit steigendem Sterbealter kontinuierlich an (Abb. 1; Zusatzmaterial online: e‑Tab. 2).

**Table 1:** 

	2001	2011	2017	Gesamt	2011, 2011, 2017 (Trend)
	*n* = 858	*n* = 1751	*n* = 2111	*n* = 4720	
	% [95 %-KI] (*n*)				*P*-Wert
Frauen	69,8	66,7	65,0	66,5	0,046
	[66,7–72,9] (599)	[64,5–68,9] (1168)	[63,0–67,0] (1373)	[65,2–67,8] (3140)	
Männer	30,2	33,3	35,0	33,5	0,046
	[27,1–33,3] (259)	[31,1–35,5] (583)	[33,0–37,0] (738)	[32,2–34,8] (1580)	
Alter, gesamt, MW ± SD	85,1 ± 7,1^b^	85,8 ± 7,1^c^	86,7 ± 6,6	86,1 ± 6,9	0,001^d^
	[84,6–85,6] (858)	[85,5–86,1] (1751)	[86,4–87,0] (2111)	[85,9–86,3] (4720)	
Alter, Frauen, MW ± SD	86,1 ± 6,8^b^	87,3 ± 6,8	88,1 ± 6,3	87,4 ± 6,6	0,001^d^
	[85,6–86,7] (599)	[86,9–87,7] (1168)	[87,7–88,4] (1373)	[87,2–87,7] (3140)	
Alter, Männer, MW ± SD	82,7 ± 7,2^b^	82,7 ± 6,6^c^	84,2 ± 6,4	83,4 ± 6,7	0,001^d^
	[81,8–83,6] (259)	[82,1–83,2] (581)	[83,7–84,7] (738)	[83,1–83,7] (1578)	
65–69 Jahre	2,6^b^	1,7	0,7	1,4	0,001^d^
	[1,5–3,7] (22)	[1,1–2,3] (30)	[0,3–1,1] (15)	[1,1–1,7] (67)	
70–74 Jahre	5,7	4,9	3,6	4,4	0,018
	[4,1–7,3] (49)	[3,9–5,9] (86)	[2,8–4,4] (75)	[3,8–5,0] (210)	
75–79 Jahre	15,4^b^	10,9	9,9	11,3	0,001^d^
	[13,0–17,8] (132)	[9,4–12,4] (191)	[8,6–11,2] (208)	[10,4–12,2] (531)	
80–84 Jahre	17,6	22,3	20,5	20,6	0,021
	[15,1–20,1] (151)	[20,4–24,2] (390)	[18,8–22,2] (432)	[19,4–21,8] (973)	
85–89 Jahre	28,6	30,5	29,0	29,5	0,480
	[25,6–31,6] (245)	[28,3–32,7] (534)	[27,1–30,9] (612)	[28,2–30,8] (1391)	
90–94 Jahre	23,9	18,7^c^	26,4	23,1	0,001^d^
	[21,0–26,8] (205)	[16,9–20,5] (327)	[24,5–28,3] (558)	[21,9–24,3] (1090)	
≥ 95 Jahre	6,3^a,b^	11,0	10,0	9,7	0,001^d^
	[4,7–7,9] (54)	[9,5–12,5] (193)	[8,7–11,3] (211)	[8,9–10,5] (458)	

*MW* Mittelwert, *SD* Standardabweichung, *n* Anzahl Verstorbener

^a^Signifikantes Testergebnis (Bonferroni korrigiert): 2001 vs. 2011

^b^Signifikantes Testergebnis (Bonferroni korrigiert): 2001 vs. 2017

^c^Signifikantes Testergebnis (Bonferroni korrigiert): 2011 vs. 2017

^d^Trendtest

**Figure 2:**
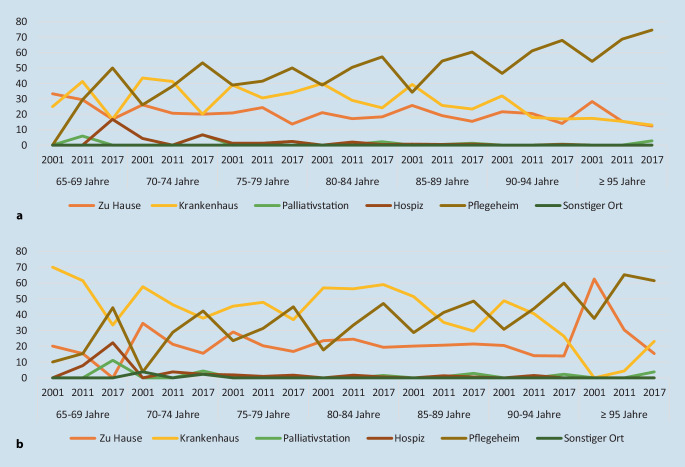

Mit 49,1 % verstarb fast die Hälfte aller Demenzerkrankten im Pflegeheim. Der zweithäufigste Sterbeort war das Krankenhaus (29,9 %), gefolgt vom häuslichen Umfeld (19,2 %). Weniger als 2,0 % der Sterbefälle ereigneten sich auf einer Palliativstation oder im Hospiz. Im zeitlichen Überblick (2001–2017) verstarben zunehmend mehr Demenzerkrankte im Pflegeheim, jedoch weniger im Krankenhaus und im häuslichen Umfeld (Tab. 2). Dieser Trend zeichnete sich bei Frauen prägnanter als bei Männern ab (Abb. 2).

**Table 2:** 

	2001	2011	2017	Total	2011, 2011, 2017 (Trend)
	*n* = 858	*n* = 1751	*n* = 2111	*n* = 4720	
	% [95 %-KI]				*P*-Wert
Häusliches Umfeld	24,0	19,7^c^	15,8^b^	19,2	0,001^d^
	[21,1–26,9]	[17,8–21,6]	[14,2–17,4]	[18,1–20,3]	
Krankenhaus	40,4^a^	29,0^c^	24,3^b^	29,9	0,001^d^
	[37,1–43,7]	[26,9–31,1]	[22,5–26,1]	[28,6–31,2]	
Palliativstation	0,0	0,3^c^	1,8	0,8	0,001^d^
	[0,0–0,0]	[0,0–0,6]	[1,2–2,4]	[0,5–1,1]	
Hospiz	0,4	0,9	0,9	0,8	0,402
	[0,0–0,8]	[0,5–1,3]	[0,5–1,3]	[0,5–1,1]	
Pflegeheim	35,2^a^	49,5^c^	57,1^b^	49,1	0,001^d^
	[32,0–38,4]	[47,2–51,8]	[55,0–59,2]	[47,7–50,5]	
Sonstiger Ort	0,0	0,0	0,0	0,0	0,393
	[0,0–0,1]	[0,0–0,0]	[0,0–0,2]	[0,0–0,1]	

^a^Signifikantes Testergebnis (Bonferroni korrigiert): 2001 vs. 2011

^b^Signifikantes Testergebnis (Bonferroni korrigiert): 2001 vs. 2017

^c^Signifikantes Testergebnis (Bonferroni korrigiert): 2011 vs. 2017

^d^Trendtest

## Diskussion

Die Studie konnte aufzeigen, dass annähernd die Hälfte aller Demenzerkrankten ≥ 65 Jahre im Pflegeheim verstirbt; jeder dritte bis vierte Sterbefall ereignet sich im Krankenhaus, jeder fünfte im häuslichen Umfeld.

Die Prävalenz einer Demenz bei Verstorbenen ≥ 65 Jahre betrug über einen 16-jährigen Beobachtungszeitraum im Mittel 14,9 % und stieg von 2001 bis 2017 von 9,2 % auf 18,0 % an. Im gleichen Zeitraum erhöhte sich das mittlere Sterbealter der Studienpopulation von 85,1 (2001) auf 86,7 (2017) Jahre, was den Prävalenzanstieg maßgeblich mitbestimmt haben dürfte. Jedoch kann dieser Effekt des Alters nicht allein den sprunghaften Anstieg der Demenzhäufigkeit zwischen 2001 (9,2 %) und 2011 (16,5 %) erklären. Die Ursache bleibt spekulativ. Möglicherweise waren Studienmitarbeiter zu Anfängen der Studie noch nicht ausreichend in dem Erkennen von Demenzfällen geschult.

Insgesamt lagen die ermittelten Prävalenzraten der Studie deutlich über der von Alzheimer Europe für 2018 angegebenen Prävalenz von 8,6 % [7]. Unterschiedliche methodische Ansätze der Falldetektion dürften diesen Unterschied erklären. Im Gegensatz zur herkömmlichen Todesursachenstatistik, die sich allein auf die Rubrik Grunderkrankung beschränkt und hier lediglich eine Erkrankung detektiert, bezog sich unsere Studie auf die Auswertung jeglicher Angaben zur Todesursache zuzüglich der Epikrise. Dadurch erhöhte sich die Chance, alle Personen mit einer Demenzerkrankung möglichst vollständig zu identifizieren.

Unsere Studienanalysen zeigen eindrücklich, dass das Pflegeheim bei Menschen mit Demenz der bestimmende Ort des Sterbens ist. Jeder zweite Verstorbene war am Lebensende in einer stationären Pflegeeinrichtung betreut worden. Dies spiegelt den intensiven pflegerischen Betreuungsbedarf dieser Patienten wider. Viele Angehörige, die demente Patienten zu Hause betreuen, stoßen im fortgeschrittenen Stadium der Erkrankung oftmals an ihre körperlichen und auch psychischen Grenzen. Die Gründe der Belastungen sind vielschichtig, u. a. hoher zeitlicher Betreuungsbedarf, Unruhezustände und gelegentlich auch aggressives Verhalten der erkrankten Personen [11].

Der Bedeutungszuwachs der Sterbeortes Pflegeheim mag neben der demografischen Entwicklung auch mit dem regionalen Ausbau von Pflegeeinrichtungen im Zusammenhang stehen. So erhöhte sich die Anzahl von Pflegeheimen von 122 (2001) auf 209 (2017). Ein Blick auf internationale Sterbeortuntersuchungen zeigt, dass das Pflegeheim auch in diesen Studien den häufigsten Sterbeort darstellt [12, 18, 24, 26, 29].

Weber et al. stellten den aktuellen Entwicklungsstand der Implementierung von Hospizkultur und Palliativkompetenz an deutschen Pflegeheimen zusammen [29]. Am Projekt nahmen 15,4 % von 10.500 bundesweit kontaktierten Pflegeeinrichtungen teil. Hier nahm das Thema Personalmangel in der Pflege einen allumfassenden Stellenwert ein. Insgesamt ließen sich Fortschritte, aber auch bestehende Defizite, die „Palliative-care“-Ausbildung von Mitarbeitern, Etablierung einer Abschiedskultur in Heimen sowie Kooperationen mit Hausärzten, Palliativnetzen und ambulanten Hospizdiensten betreffend, feststellen. Eine Palliative-care-Qualifikation (40 h Basiskurs/160 h Spezialkurs) konnten vorweisen: Leiter der Einrichtungen (12,3 %/11,2 %), Pflegedienstleitungen (16,5 %/20,3 %), Pflegefachkräfte (13,8 %/14,6 %). Einrichtungsleiter selbst schätzten die erreichte Palliativkompetenz im Heim wie folgt ein: sehr gut: 9 %, gut 52 %, befriedigend 33 %, ausreichend 5 %, mangelhaft bis ungenügend 1 %.

Den zweithäufigsten Sterbeort stellt in unserer Untersuchung das Krankenhaus dar. Hier ergab sich ein abnehmender Trend. Einen möglichen Erklärungsansatz könnte der wachsende Kostendruck im Krankenhaussektor liefern. So reduzierte sich mit Einführung der DRG im Jahre 2003 die stationäre Liegedauer in Kliniken deutlich, bei gleichzeitiger Verlagerung medizinischer Leistungen in den vor- und nachstationären Sektor [13]. Die Gründe für eine Klinikeinweisung demenzerkrankter Personen sind vielschichtig. Oft bedingen Schluckstörungen mit konsekutiv erhöhtem Aspirationsrisiko, Infektionen des Urogenitaltraktes sowie Stürze – u. a. im Kontext eines Frailty-Syndroms, einer Apraxie oder medikamentöser Nebenwirkungen – eine Klinikbehandlung [5, 22, 23]. Untersuchungen verweisen darauf, dass der Transfer ins Krankenhaus für Demenzerkrankte besonders belastend ist. So können Verwirrtheits- und Unruhezustände aggravieren [8].

Wir beobachteten ebenso einen abnehmenden Trend häuslicher Sterbefälle (24,0 % (2001), 19,7 % (2011), 15,8 % (2017)). Im internationalen Vergleich liegen die Studienresultate im Mittelfeld. Soziale Veränderungen in der Gesellschaft, weg von der pflegenden (Groß‑)Familie, hin zu mehr alleinlebenden Personen, mögen zu dieser Entwicklung beitragen [27]. Zwar werden nach wie vor die meisten Pflegebedürftigen von Familienangehörigen gepflegt [3], jedoch ändern sich die Rahmenbedingungen. Abnehmende Geburtenzahlen, zunehmende Berufstätigkeit pflegender Angehöriger und entfernte Wohnortverhältnisse zwischen erwachsenen Kindern und Eltern sind nur einige Faktoren, die Pflege durch Angehörige im privaten Umfeld erschweren.

Das Hospiz nimmt bei Personen mit Demenz als Sterbeort eine untergeordnete Rolle ein. Dies mag v. a. dadurch begründet sein, dass – historisch begründet – hauptsächlich Tumorpatienten im Hospiz betreut werden. Auch spielt die Tatsache eine Rolle, dass ein Wechsel vom Pflegeheim in ein Hospiz in der Realität kaum vorkommt, da Krankenkassen eine Heimversorgung als adäquat und ausreichend ansehen. Weniger mag der Erkrankungsverlauf einen Grund darstellen. Zwar verläuft der Krankheitsprozess zumeist langsam, was im Widerspruch zur Vorgabe einer Hospizverlegung steht, nur dann Patienten ins Hospiz aufzunehmen, wenn die ärztliche Einschätzung der Lebenserwartung weniger als 3 bis 6 Monate beträgt, jedoch begrenzt sich die Lebenserwartung im fortgeschrittenen Stadium der Erkrankung häufig nur noch auf wenige Wochen. Eine angemessene Aus- und Fortbildung des Hospizpersonals im Umgang mit demenziell Erkrankten wäre insgesamt wünschenswert.

Das Thema „end-of-life care“ von Demenzerkrankten stellt das Gesundheitssystem vor erhebliche Herausforderungen. Hierzu hat die Europäische Gesellschaft für Palliative Care (EAPC) in einem Positionspapier Stellung genommen [28]. Als wesentliche Elemente einer guten Palliativversorgung bei dementen Personen wurden u. a. eine personenzentrierte Betreuung, eine gute Symptomenkontrolle, eine Behandlungskontinuität, das Festlegen von Therapiezielen, das Vermeiden belastender und/oder vergeblicher Therapien, Angebote psychosozialer und spiritueller Unterstützung, die Betreuung und der Einbezug pflegender Angehöriger sowie eine adäquate Ausbildung des Behandlungsteams benannt.

Vonseiten des Gesetzgebers wurde eine nationale Demenzstrategie auf den Weg gebracht, mit dem Ziel, die Situation von Menschen mit einer Demenz in allen Lebensbereichen nachhaltig zu verbessern [4]. So ist u. a. beabsichtigt, Beratungen zu hospizlichen und palliativmedizinischen Angeboten und zur vorausschauenden Gesundheitsplanung zu fördern und Kooperationen mit ambulanten Hospizdiensten und regionalen palliativmedizinischen Leistungsanbietern zu stärken. Es bleibt abzuwarten, inwieweit die vereinbarten Ziele auch tatsächlich in der alltäglichen Praxis Umsetzung finden werden.

### Stärken und Schwächen

Die vorliegende Studie stellt die bis dato umfangreichste Sterbeortuntersuchung bundesweit dar. Da sich die Analysen auf ausgewählte westfälische Regionen bezogen, können die Ergebnisse jedoch nicht als repräsentativ gelten. Die Sterbeortangaben können als valide betrachtet werden. Totenscheine des Krankenhauses waren fast immer mit einem Institutsstempel versehen, Hospize stets und Pflegeheime überwiegend durch ihre postalische Anschrift gekennzeichnet.

Es ist bekannt, dass die Demenz von ärztlicher Seite oftmals nicht als Grunderkrankung im Totenschein dokumentiert wird [21]. Die Folge ist eine Unterschätzung der Todesursache Demenz. Wir versuchten, diesem Problem entgegenzuwirken, indem wir jedwede ärztliche Angabe zur Todesursache auswerteten. Angaben zur Zeitspanne zwischen Krankheitsbeginn und Tod lagen nur vereinzelt vor. Dementsprechend fehlten Informationen zur Erkrankungsdauer. Auch ließen die Daten keine Aussage zur Schwere der Erkrankung zu.

## Fazit für die Praxis

- Annähernd die Hälfte der älteren Menschen mit einer Demenz (≥ 65 Jahre) verstirbt im Pflegeheim. Jeder dritte bis vierte Sterbefall ereignet sich im Krankenhaus, jeder fünfte im häuslichen Umfeld.
- Palliativstationen und Hospize spielen als Sterbeorte bei Demenzerkrankten eine untergeordnete Rolle.
- Im zeitlichen Verlauf (2001–2017) zeigt sich, dass Menschen mit einer Demenz vermehrt im Pflegeheim versterben, hingegen Sterbefälle im Krankenhaus sowie häuslichem Umfeld rückläufig sind.

## Supplementary Information






## Abkürzungen

DRG: Diagnosebezogene Fallgruppen
EAPC: Europäische Gesellschaft für Palliative Care
ICD: Internationale statistische Klassifikation der Krankheiten und verwandter Gesundheitsprobleme
