# Supplementary material for: Place of death of older people with dementia: Epidemiological data from an observational study of places of death in Germany (2001, 2011, 2017)
Source: Z Gerontol Geriatr. 2021 Sep 30;55(8):673–9. [Article in German] doi: 10.1007/s00391-021-01976-7 (PMC9726759; doi:10.1007/s00391-021-01976-7)
Supplement: Supplementary file 1 [file 391_2021_1976_MOESM1_ESM.docx]

**e-Tabelle 1** Strukturdaten der Studienregion – stratifiziert nach Untersuchungszeitpunkt

|  | **2001** | **2011** | **2017** |
| --- | --- | --- | --- |
| **Einwohnerzahl (in 1000)** | 1.282 | 1.298 | 1.343 |
| **Einwohner (in 1000) ≥65 Jahre** | 245 (19,1%) | 295 (22,7%) | 322 (24,0%) |
| **Krankenhäuser** | 30 | 28 | 26 |
| **Krankenhausbetten** | 9.985 | 9.805 | 9.865 |
| **Krankenhausbetten pro 1 Mio.** | 7.789 | 7.554 | 7.346 |
| **Palliativstationen** | 0 | 7 | 13 |
| **Palliativbetten** | 0 | 40 | 79 |
| **Palliativbetten pro 1 Mio.** | 0 | 30,8 | 58,8 |
| **Hospize** | 3 | 6 | 6 |
| **Hospizbetten** | 27 | 50 | 52 |
| **Hospizbetten pro 1 Mio.** | 21.1 | 38.5 | 38.7 |
| **Pflegeheime** | 122 | 166 | 209 |
| **Pflegeheimbetten** | 9.592 | 11.276 | 12.991 |
| **Pflegeheimbetten pro 1 Mio.** | 7.482 | 8.687 | 9.673 |
| **Ambulante palliativmedizinische Konsiliardienst (PKD)** | 0 | 5 | 6 |
