# Supplementary material for: Place of death of older people with dementia: Epidemiological data from an observational study of places of death in Germany (2001, 2011, 2017)
Source: Z Gerontol Geriatr. 2021 Sep 30;55(8):673–9. [Article in German] doi: 10.1007/s00391-021-01976-7 (PMC9726759; doi:10.1007/s00391-021-01976-7)
Supplement: Supplementary file 2 [file 391_2021_1976_MOESM2_ESM.docx]

**e-Tabelle 2.** Anteil Verstorbener mit einer Demenz (≥ 65 Jahre) – stratifiziert nach Geschlecht und Sterbejahr

|  | **2001** | | | **2011** | | | **2017** | | |
| --- | --- | --- | --- | --- | --- | --- | --- | --- | --- |
|  | **Männer** | **Frauen** | **Gesamt** | **Männer** | **Frauen** | **Gesamt** | **Männer** | **Frauen** | **Gesamt** |
|  | **N=3.895** | **N=5.402** | **N=9.303** | **N=4.819** | **N=5.781** | **N=10.603** | **N=5.388** | **N=6.337** | **N=11.725** |
|  | **% [95%-KI] (n)** | | | | | | | | |
| **Verstorbene mit Demenz** | 6,6  [5,8-7,4]  (259) | 11,1  [10,3-11,9]  (599) | 9,2  [8,6-9,8]  (858) | 12,1  [11,2-13,0]  (581) | 20,2  [19,2-21,2]  (1.168) | 16,5  [15,8-17,2]  (1.751) | 13,7  [12,8-14,6]  (738) | 21,7  [20,7-22,7]  (1.373) | 18,0  [17,3-18,7]  (2.111) |

Fehlende Angaben zum Geschlecht (2001: n=6; 2011: n=3; 2017: n=0)
